# Predicting conversion from clinically isolated syndrome to multiple sclerosis–An imaging-based machine learning approach

**DOI:** 10.1016/j.nicl.2018.11.003

**Published:** 2018-11-05

**Authors:** Haike Zhang, Esther Alberts, Viola Pongratz, Mark Mühlau, Claus Zimmer, Benedikt Wiestler, Paul Eichinger

**Affiliations:** a Department of Diagnostic and Interventional Neuroradiology, Klinikum rechts der Isar, Technical University of Munich, Ismaninger Strasse 22, 81675 Munich, Germany; b Department of Neurology, Klinikum rechts der Isar, Technical University of Munich, Ismaninger Strasse 22, 81675 Munich, Germany; c TUM-NIC, NeuroImaging Center, Klinikum rechts der Isar, Technical University of Munich, Ismaninger Strasse 22, 81675 Munich, Germany

**Keywords:** Multiple sclerosis, Clinically isolated syndrome, MRI, Machine learning, CIS, clinically isolated syndrome, MS, Multiple Sclerosis, CDMS, clinically definite Multiple Sclerosis, SVR, surface-volume-ratio

## Abstract

Magnetic resonance imaging (MRI) scans play a pivotal role in the evaluation of patients presenting with a clinically isolated syndrome (CIS), as these may depict brain lesions suggestive of an inflammatory cause. We hypothesized that it is possible to predict the conversion from CIS to multiple sclerosis (MS) based on the baseline MRI scan by studying image features of these lesions.

We analyzed 84 patients diagnosed with CIS from a prospective observational single center cohort. The patients were followed up for at least three years. Conversion to MS was defined according to the 2010 McDonald criteria. Brain lesions were segmented based on 3D FLAIR and 3D T1 images. We generated brain lesion masks by a computer assisted manual segmentation. We also generated a set of automated segmentations using the Lesion Segmentation Toolbox for SPM to assess the influence of different segmentation methods. Shape and brightness features were automatically calculated from the segmented masks and used as input data to train an oblique random forest classifier. Prediction accuracies of the resulting model were validated through a three-fold cross-validation.

Conversion from CIS to MS occurred in 66 of 84 patients (79%). The conversion or non-conversion was predicted correctly in 71 patients based on shape features derived from the computer assisted manual segmentation masks (84.5% accuracy). This predictor was more accurate than predicting conversion using dissemination in space at baseline according to the 2010 McDonald criteria (75% accuracy). While shape features strongly contributed to the accuracy of the predictor, including intensity features did not further improve performance.

As patients who convert to definite MS benefit from early treatment, an early classification model is highly desirable. Our study shows that shape parameters of lesions can contribute to predicting the future course of CIS patients more accurately.

## Highlights

• A random forest tool can help to identify patients who convert from clinical isolated syndrome into multiple sclerosis (MS).
• The classifier is driven by shape features of lesions in the first MR scan.
• The found shape features reflect the typical ovoid growth of MS lesions.

## 1 Introduction

A clinically isolated syndrome (CIS) is defined as a single episode of neurological symptoms suggestive of an inflammatory demyelinating disease of the central nervous system. It is the initial presentation of multiple sclerosis (MS) for many patients (Miller et al., 2012). Magnetic resonance imaging (MRI) plays a key role in the evaluation of patients with CIS. It may depict brain lesions which potentially substantiate the suspicion of a chronic inflammatory disease. In fact, according to the McDonald criteria 2010 (Polman et al., 2011), dissemination in space and dissemination in time can be proven by MR findings. Together with the onset of clinical symptoms, this can be used to diagnose MS. Previous studies have confirmed that patients presenting with CIS and abnormal MRI findings in baseline scans have higher risk for conversion to MS (Zhang and Hou, 2013) and this risk is associated with the number of lesions (Fisniku et al., 2008; Tintoré et al., 2006).

However, not all patients with CIS develop multiple sclerosis: 20% of CIS patients do not convert after twenty years even with abnormal findings in baseline MR images (Fisniku et al., 2008). Three years after the first clinical onset, according to earlier studies 31–44% of the CIS patients experience new attacks which define clinically definite MS (CDMS) (Liu et al., 2011; Miller et al., 2012; Rocca et al., 2008; Tintoré et al., 2000). Note, that it is possible that patients are diagnosed with MS according to the 2010 McDonald criteria but not with CDMS as defined above. This is the case, when a follow up MRI scan of a CIS patient shows new lesions such that the criteria of dissemination in space are fulfilled but the patient has not experienced a second clinical attack in the meantime. This is referred to as “radiologically definite MS”. For radiologically definite MS, reported conversion rates are higher than those for CDMS (Chard et al., 2011; Gaetani et al., 2017).

By identifying reliable parameters which predict conversion, patients with a CIS who profit from an early treatment could be selected more reliably. For example, treatment with β-interferon and glatiramer acetate was shown to delay conversion to MS (Comi et al., 2009; Comi et al., 2001; Jacobs et al., 2000).

MRI scans generate a large amount of data. Only a small part of it is used in clinical routine because the images are only studied visually. In the past few years, strategies to analyze large collections of data have emerged, as well as algorithms for solving classification and prediction tasks. Machine learning has become a promising way to process medical images. In the field of neurology and neuroradiology, such techniques have been applied to various classification tasks, such as assessing epilepsy (Song et al., 2012), pre-symptomatic Huntington's disease (Klöppel et al., 2009), gliomas WHO Grade II and III (Eichinger et al., 2017) and Alzheimer's disease (Escudero et al., 2013; Klöppel et al., 2008). Machine learning has also been used in MS to distinguish MS patients from healthy controls (Yoo et al., 2018), different disease courses in MS patients (Ion-Margineanu et al., 2017; Zhao et al., 2017) and also to predict conversion in CIS patients. The latter was addressed by (Wottschel et al., 2015), who included clinical and basic lesion features, and by (Kitzler et al., 2018), who analyzed lesion myelination.

In this study, we investigated how a machine learning tool can help to identify CIS patients who convert to MS. To this end, we focussed on lesion features in baseline MRI, in particular such features that describe shape and brightness, and assessed the performance of machine learning classifiers based on these features.

## 2 Methods

### 2.1 Subjects

This study includes 84 patients who initially presented with CIS, i.e. showed symptoms suggestive of an inflammatory central nervous disease without fulfilling the 2010 McDonald criteria for MS. All patients were part of a single center prospective observational cohort, which was approved by the local institutional review board, and written informed consent was obtained. All patients received a baseline MRI scan during primary clinical work-up. These baseline scans were acquired between 2009 and 2013. Patients were followed up regularly for a period of at least three years. An MRI scan after three years as well as clinical evaluation was used to determine whether conversion into MS had occurred. MS was defined according to the 2010 McDonald criteria. In particular, besides demonstrating dissemination in time by a clinical relapse, the occurrence of new MRI lesions sufficed to prove dissemination in time as well.

### 2.2 MRI acquisition and processing

All MR images were acquired using a 3 Tesla MR scanner (Achieva, Philips Healthcare, Best, the Netherlands). All MR scans contained a 3D Fluid attenuated inversion recovery (FLAIR) sequence and a 3D T1 sequence, which were used for this study. The imaging parameters were as follows:

FLAIR: Acquired voxel size, 1.03 × 1.03 × 1.5 mm^3^; acquisition matrix, 224 × 154; field of view, 230; TR, 10000 ms; TE, 140 ms; TSE factor, 20; number of slices, 96; acquisition time, 5 min; plane, axial.

T1: Acquired voxel size, 1 × 1 × 1 mm^3^; acquisition matrix, 240 × 240; field of view, 240; TR, 9 ms; TE, 4 ms; number of slices, 170; acquisition time, 6 min; plane, sagittal.

Based on the FLAIR and T1 weighted images, two sets of lesion segmentation masks were generated. One set was acquired by computer assisted manual segmentation using BrainSeg3D (Lesjak et al., 2018). For this, a neuroradiologist (PE, 6 years of experience) identified and marked lesions on axial reformations of the FLAIR images. The precise borders of these lesions within one slice were then delineated by the segmentation tool, leaving the option to manually readjust the segmentation. This segmentation set was used as the main tool during further analysis. A second set of lesions masks was obtained with the Lesion Segmentation Tool (LST) (Schmidt et al., 2012), version 2.0.1, which was designed for the Statistical Parametric Mapping package for MATLAB (SPM 12, Wellcome Trust Centre for Neuroimaging; MATLAB and Statistics Toolbox Release 2016b, The MathWorks, Inc., Natick, Massachusetts, United States). This provided a fully automated segmentation set, using LST's lesion probability algorithm (initial threshold 0.3). The second set of segmentations was acquired to assess the dependence of our classifier on the chosen segmentation method.

### 2.3 Classification analysis

For each lesion individually, the single lesion volume, intensity features (skewness, kurtosis and entropy of intensity histograms) and shape features (surface area, sphericity, surface-volume-ratio (SVR)) were calculated automatically. Moreover the total number of lesions and the total lesion volume were noted. The lesion surface area (A) was approximated through a marching cubes algorithm implemented in SciKit-Image. From the surface area and volume (V; given as *n* ∗ *voxel volume*, with n being the number of voxels in the lesion), sphericity was calculated as $\frac{\sqrt[{3}]{36\mathrm{\pi V}2}}{A}$.

The random forest algorithm, which is described below, requires a feature vector of the same length for each patient. We therefore could not include the single lesion parameters as described above because patients differed in lesion numbers. To achieve a uniform parameter set in each patient, we generated descriptive statistics for volume, intensity and shape features. Since merely averaging over all lesions in a patient would neglect information on lesion heterogeneity, we included the minimum, maximum, mean and standard deviation of each feature with respect to all lesions in this patient. In addition, we included the total lesion volume, calculated as the sum of the single lesion volumes, and lesion count as further elements of the vector.

Random forest algorithms are an ensemble learning strategy based on generating a forest, i.e. a collection of many uncorrelated decision trees (Breiman, 2001). Generating uncorrelated trees is achieved by repeatedly and randomly drawing samples and features with replacement. As opposed to the traditional threshold-based random forest, which uses univariate models at each node, an oblique random forest explicitly learns the optimal split between two groups using linear multivariate models. This approach has been shown to further improve robustness and accuracy (Menze et al., 2011), especially in the case of correlated features. To predict conversion into MS, we generated three oblique classification random forest models using the “obliqueRF” package in R 3.4 (The R Foundation for Statistical Computing, Vienna, Austria): (i) A model based on intensity features, (ii) a model based on shape features and (iii) a final model including both intensity and shape features. Lesion count and lesion volume were included as features in each of these models. The hyperparameters “mtry” (number of variables tested in each node; tested with 3, sqrt(number of variables) and 7 variables) and “ntree” (number of trees generated, tested with 100, 200 and 300 tress) were optimized on the out-of-bag error, i.e. by testing on the samples which were not randomly drawn for this tree during forest generation. The coefficients of the split were found in a L2 constraint least squares regression, tested for various regularization coefficients.

### 2.4 Data and statistical analysis

Model performance was validated by three-fold cross-validation. For that, the study collective was randomly divided in three subsets. One of these subsets was not used during the training process so that the algorithm was adjusted to only two thirds of the whole study cohort. The remaining third was then used as validation set on which the algorithm was tested. This was done three times, so that every subset functioned as validation set once and every subject was within the validation set once. Only cross-validated performance measures are reported. Using a bootstrapping approach (with 100 iterations), we calculated feature importance scores to identify the features most important for classification from the oblique random forest. Feature importance counts how often a variable was deemed relevant when chosen for a split at a node. This is achieved by a logistic regression model which is employed at the nodes. The importance value is increased by 1 if a variable leads to a logistic regression model with p < 0.05 at a node, and decreased by 1 otherwise.

Besides the described random forest models, another predictive model was also obtained based on the criteria for dissemination in space according to the 2010 McDonald criteria, analogously to (Filippi et al., 2018). For this model, only the criteria of dissemination in space have to be assessed. If they are fulfilled at baseline, then the prediction is set to “conversion”. This model was included as a benchmark for the computer based models described above.

Statistical analysis was performed using MATLAB 2016b (MATLAB and Statistics Toolbox Release 2016b, The MathWorks, Inc., Natick, Massachusetts, United States). Demographic data of non-converters and converters were compared using a Pearson's chi-square test (gender) and a two tailored *t*-test (age). EDSS values at baseline and after 3 years were compared between the groups by a Mann-Whitney *U* test. For each group, the EDSS baseline value and the EDSS value after 3 years were compared by a Wilcoxon signed rank-sum test. The results from the different prediction approaches as defined above were expressed as confusion matrices and the following statistical measures were derived: accuracy, sensitivity, specificity, positive and negative predictive value. Moreover, we also calculated the balanced accuracy (Brodersen et al., 2010) together with the posterior probability interval for α = 0.05 using the MATLAB tools provided by Brodersen et al. and the diagnostic odds ratio (Glas et al., 2003). Confidence intervals were calculated for accuracy, sensitivity and specificity as Clopper-Pearson confidence intervals. Those for positive and negative predictive values were calculated as standard logit confidence intervals (Mercaldo et al., 2007). The confidence interval for the diagnostic odds ratio was calculated according to (Glas et al., 2003). The random forest classifier was compared with the classifier based on the 2010 McDonald criteria by using McNemar's test.

## 3 Results

### 3.1 Subjects

Our cohort included 58 female and 26 male patients. None of the 84 patients were excluded in the subsequent analyses. During the three years of follow up, 66 patients converted to MS (79%) while 18 patients did not. From the 66 patients who converted to MS, 33 had a second clinical attack defining CDMS and in 33 patients, conversion was detected based on radiological criteria. The relevant patient characteristics are summarized in Table 1. The two groups of converters and non-converters were not significantly different in terms of gender, age, EDSS at baseline or after 3 years follow-up. Within the groups, neither converters nor non-converters showed a significant change in EDSS during the observation period (p = 0.87 and p = 0.29, respectively; Wilcoxon signed rank-sum test). One patient received Interferon Beta, one received steroids and one received plasmapheresis prior to the baseline MRI scan. Apart from those three patients, the remaining 81 patients did not receive any therapy prior to their baseline scan.

**Table 1:** Patient characteristics.

	Non converter	Converter	
Gender	7 men	18 men	Pearson Chi Square,
	11 women	47 women	p = 0,411
Age	Mean = 44,44	Mean = 41,89	2-tailed *t*-Test,
	STD = 11,21	STD = 8808	p = 0,308
EDSS at baseline	Median = 1	Median = 1	Mann-Whitney *U* test,
	Range 0–2.5	Range 0–6	p = .56
EDSS after 3 years	Median = 0	Median = 1	Mann-Whitney U test,
	Range 0–2.5	Range 0–6.5	*p* = .08
Mean lesion volume (mm^3^)	Mean = 71	Mean = 135	Mann-Whitney U test,
	Range 22–314	Range 22–671	p = .0013

### 3.2 Classification results using brightness and shape features

To assess prediction accuracies of the random forest models and compare them to those achieved by the classifier based on the 2010 McDonald criteria, we calculated the confusion matrix for each predictive model. Table 2 shows those confusion matrices.

**Table 2:** Confusion matrices for different predictive models assessed in this study: (a) 2010 McDonald criteria (dissemination in space (DIS) yes/no); (b) intensity based random forest classifier using computer assisted manual segmentations; (c) shape based random forest classifier using computer assisted manual segmentations; (d) shape based random forest classifier using automated segmentations from LST.

a) McDonald 2010 (DIS)	Non-conversion	Conversion
Predicted non-conversion	4	4
Predicted conversion	14	62

b) Intensity-based model	Non-conversion	Conversion
Predicted non-conversion	11	25
Predicted conversion	7	41

c) Shape-based model	Non-conversion	Conversion
Predicted non-conversion	9	4
Predicted conversion	9	62

d) Shape-based model (LST)	Non-conversion	Conversion
Predicted non-conversion	6	3
Predicted conversion	12	63

Table 3 shows statistical measures derived from the confusion matrices. We also included balanced accuracy (Brodersen et al., 2010) and the diagnostic odds ratio (Glas et al., 2003), which are designed for unbalanced cohorts, such as the one in this study with a conversion rate of 79%. The shape-based random forest classifier (with 300 tress and 3 variables tested at each split) achieved the highest accuracy. Particularly balanced accuracy and diagnostic odds ratio are markedly higher than for prediction according to the McDonald criteria 2010. Further comparing the classifier based on shape features with prediction of conversion according to the McDonald criteria 2010 by McNemar's test also showed a significantly higher predictive accuracy of the random forest classifier (p = 0.03).

**Table 3:** Statistical measures derived from the confusion matrices in Table 2. Intervals are 95%-confidence intervals, except for balanced accuracy, where the posterior probability interval for the level 0.05 is given (as defined in (Brodersen et al., 2010)). PPV, positive predictive value; NPV, negative predictive value; DOR, diagnostic odds ratio.

	Mc Donald 2010 (DIS)	Intensity-based model	Shape-based model	Shape-based model (LST)
Accuracy	0.79 (0.68–0.87)	0.62 (0.51–0.72)	0.85 (0.75–0.91)	0.82 (0.72–0.90)
Sensitivity	0.94 (0.85–0.98)	0.62 (0.49–0.74)	0.94 (0.85–0.98)	0.95 (0.87–0.99)
Specificity	0.22 (0.06–0.48)	0.61 (0.36–0.83)	0.50 (0.26–0.74)	0.33 (0.13–0.59)
PPV	0.81 (0.77–0.85)	0.85 (0.76–0.92)	0.87 (0.81–0.91)	0.84 (0.79–0.87)
NPV	0.50 (0.22–0.78)	0.31 (0.21–0.42)	0.69 (0.44–0.87)	0.67 (0.36–0.88)
Balanced Accuracy	0.58 (0.50–0.70)	0.62 (0.49–0.72)	0.72 (0.60–0.82)	0.64 (0.54–0.76)
DOR	4.43 (0.99–19.89)	2.58 (0.88–7.51)	15.50 (3.93–60.98)	10.50 (2.30–47.87)

A second model based on shape features, but using lesion masks from the automated segmentation with LST, performed similar to the one using the computer-assisted manual segmentations.

To compare computer assisted manual and fully automated segmentations, we assessed the correlation between the three most important features, and found a high correlation for “mean volume” (Pearson's *r* = 0.79, p < 0.0001), “minimum sphericity” (Pearson's *r* = 0.42, p < 0.0001) and “minimum SVR” (Pearson's *r* = 0.88, p < 0.0001).

### 3.3 The three most relevant shape features

**Fig. 1:**
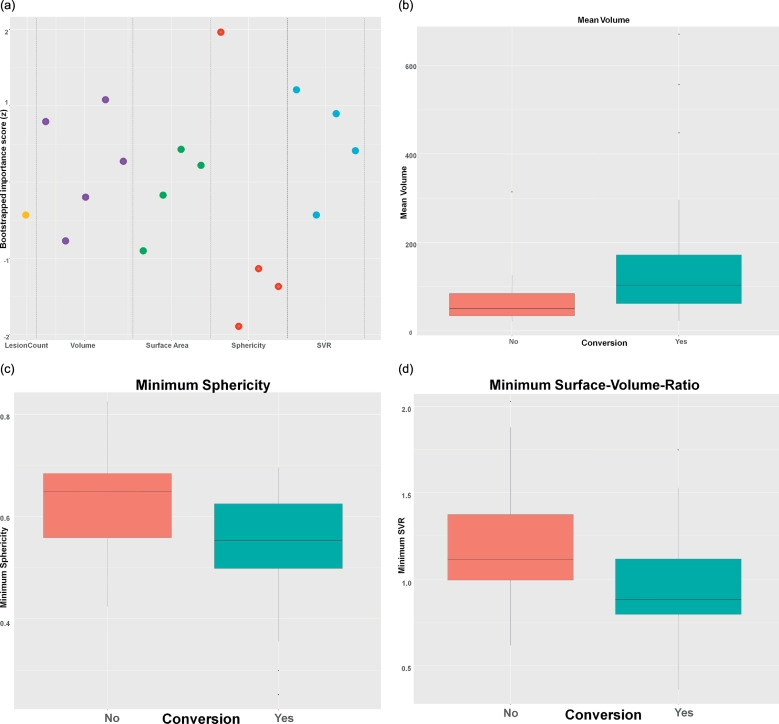
Shape features most relevant for predicting conversion. (a) Bootstrapped importance plot, where each dot represents a feature (i.e. min, max, mean and std. of the respective measure, for volume also total lesion volume). The higher a feature, the more important it is for prediction. (b-d) Boxplot diagrams of the three most important features, which scored importance scores >1: mean volume (b), minimum sphericity (c) and minimum surface-volume-ratio (d), separated by converters (turquoise) and non-converters (red).

To explore the relative influence of shape features on the prediction of conversion, we calculated importance scores using a bootstrapping approach (Fig. 1a). While all features input into the classifier will contribute to the final prediction, we identified three features that had the highest importance for the final vote: Mean lesion volume (Fig. 1b), minimum sphericity (Fig. 1c) and minimum surface-volume-ratio (Fig. 1d). As expected, minimum sphericity and minimum surface-volume-ratio had a significant positive correlation (Spearman's rho = 0.53, p < 0.001).

**Fig. 2:**
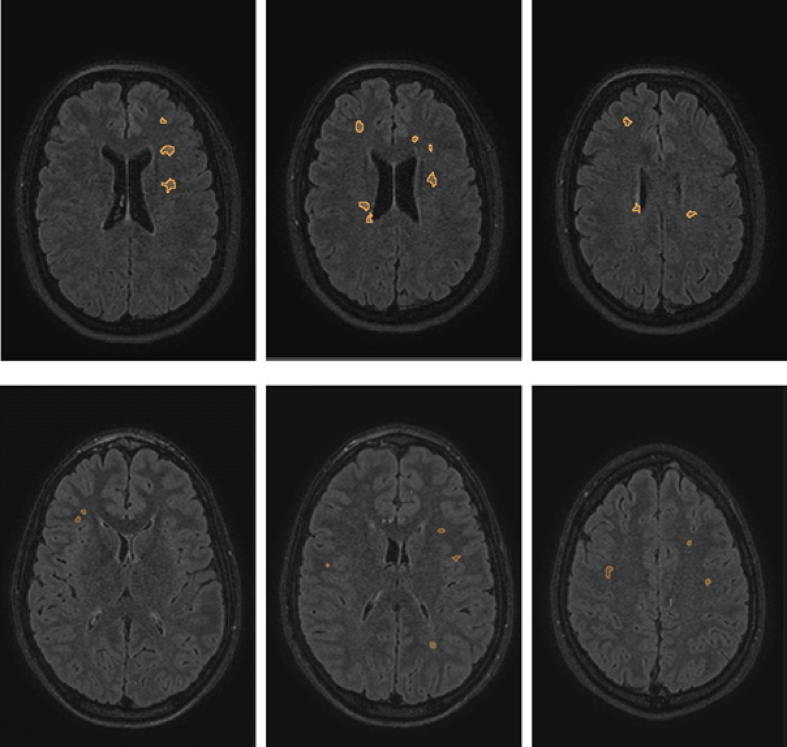
Illustrative example images with overlaid lesion masks of a patient who converted to MS (upper row) and another patient who did not convert (lower row). These examples very prominently represent the lesion features with the best discriminative potential. Note the larger, less round lesions in the upper row example. The numerical values for the converter (top row) were: mean volume, 101 mm^3^; mean sphericity, 0.78; mean SVR, 1.64; the non-converter (bottom row) showed the following values: mean volume, 33.8mm^3^; mean sphericity, 0.927; mean SVR, 2.1.

Looking into the distribution of these features, the classifier found that lesions in converters were on average larger and appeared less round (as expressed by a smaller minimum sphericity) compared to lesions in non-converters. Interestingly, lesion count was not found to be of high importance. Fig. 2 shows illustrative examples of a converter and a non-converter, which demonstrate the differences in lesion appearance.

## 4 Discussion

In this study, we developed a random forest classifier based on lesion features in the baseline MRI to differentiate patients converting from CIS to MS from those who do not. While shape features demonstrated a high discriminative potential, intensity features did not contribute to improving this classifier. We demonstrated that both computer assisted manual and fully automatic segmentations yield shape features that can be used to construct an accurate classifier. This could point towards the use of shape features as more generalizable features. Moreover, this finding can help to transfer our technique to larger cohorts, for which manual segmentation becomes less feasible due to the time required to generate the lesion masks.

The resulting classifier was more accurate than predicting conversion using the dissemination in space criteria from the 2010 McDonald criteria. In particular, this holds true for measures designed for unbalanced cohorts like balanced accuracy and diagnostic odds ratio. The random forest classifier resulted in a sensitivity of 94% and specificity of 50% as opposed to 94% and only 22%, respectively for the 2010 McDonald criteria. The results for the 2010 McDonald criteria closely match a recent study which assessed the performance of MRI criteria to predict conversion in a large cohort (Filippi et al., 2018) but did not employ machine learning techniques.

In our study, we determined MS according to the 2010 McDonald criteria, which also include radiological criteria to establish dissemination in space and time. This differs from other studies where only conversion to CDMS, i.e. the occurrence of a second clinical attack, is considered. Hence, the reported conversion rate of 79% in our cohort is noticeably higher than in previous studies. Note, that the rate of CDMS in our cohort is 39% which is comparable to other studies like those mentioned in the introduction. Also, other studies that assessed conversion into radiologically definite MS (which is equivalent to our approach) demonstrated conversion rates comparable to those found in this study (Chard et al., 2011; Gaetani et al., 2017).

Comparing our approach to other studies with a similar aim, we focussed mainly on imaging features that describe lesion properties. In an earlier study (Wottschel et al., 2015), basic clinical features were combined with features of lesion localization to predict conversion. Opposed to our study, only basic intensity features (average lesion PD- and T2-intensities) have been employed. Other studies used advanced MR techniques, such as measuring myelin water fraction in white matter (Kitzler et al., 2018) or MR spectroscopy (Ion-Margineanu et al., 2017). While these methods can provide new insight into how MS patients can be assessed with MR imaging, they are not commonly used. In comparison, our study only relies on FLAIR and T1-weighted images which are considered to be part of a standard imaging protocol in MS (Traboulsee et al., 2016).

Further assessing the relative contribution of each feature, we found that mean lesion volume, minimum sphericity and minimum SVR were most important for the performance of our classifier: Lesions found in patients converting to MS are on average bigger and less spherical. We hypothesize that this difference in size and shape reflects the different origin of lesions in converters: Inflammation in MS is considered to most commonly occur along veins (Tallantyre et al., 2008; Tan et al., 2000). The presence of a central vein within a lesion can be used to differentiate MS from other causes of white matter hyperintensities (Cortese et al., 2018; Maggi et al., 2018). This might favour the development of more elongated, less spherical inflammatory lesions in MS converters. The typical “Dawson finger configuration” of MS lesions seen in sagittal images in fact reflects this and is an example of how neurologists and neuroradiologists use shape information to classify lesions. On the other hand, the lesions found in patients who do not convert to MS may be caused by a different pathomechanism and therefore differ in shape from the inflammatory lesions found in patients converting to MS.

Intensity features have long been used in image analysis. However, analysis of MR intensities is impeded by several obstacles, including bias-field inhomogeneities and variations in scanner equipment and sequences. Moreover, intensity values may change over time especially early after lesion formation when the acute inflammation wears off. Such features therefore tend to be poorly generalizable beyond the data set based for which they were designed. Shape features, on the other hand, are to some extent invariant against the input data. In comparison to brightness features, they do not rely on absolute intensity values and are robust against bias field distortions. Therefore, these features are more comparable across different scanners. Within our cohort, we found no meaningful contribution of intensity features to prediction accuracy. In fact, a model solely based on intensity features performed worse than both the shape-based and McDonald-based model.

Our study has several limitations: Shape features contributed most to our model. However, the minimum diameter of a lesion that can be depicted is determined by the voxel size of the scan. Therefore, shape features of small lesions can be influenced by the spatial resolution of a scan. Given a minimum size threshold for a lesion of 3 mm as proposed in the MAGNIMS- and McDonald-Criteria (Filippi et al., 2016; Polman et al., 2011), 3D sequences with high spatial resolution (like those used in this study) are not very prone to these effects. However, extracting reliable features for small lesions may become impossible when 2D images are used, which impedes the possibility of transferring our approach to 2D images.

Complex machine learning algorithms are prone to overfitting and unvalidated accuracies will be biased. Given the size of our cohort, we chose a three-fold cross-validation to validate all predictions made by our classifiers. While k-fold cross-validation has been shown to give more realistic estimates of prediction accuracy than leave-one-out-cross-validation, an independent validation in a previously unseen cohort of patients would be preferable.

We selected the features which were used to develop the classifier. How other image features like texture features or more advanced, deep-learned features could contribute to prediction accuracy is therefore unclear. However, the size of our dataset precludes extensive testing of large feature vectors. We therefore limited our analysis to pre-defined features mentioned above.

In general, we limited on a narrow spectrum of features to specifically assess the contribution of lesion characteristics to classification performance. How other clinical and paraclinical parameters like age, gender, baseline EDSS (these were already covered in (Wottschel et al., 2015)), intrathecal synthesis of oligoclonal bands (Tintoré et al., 2008) or inflammatory cerebrospinal fluid (Ruet et al., 2014) can be used to further improve prediction accuracy remains to be investigated in future studies. Moreover, the disease course in MS patients is highly individual. Therefore, rather than only predicting conversion in CIS patients, it appears an promising goal to predict different disease courses and identify those patients who are likely to profit most from early treatment, taking into account different patterns of disease activity in MS patients.

## 5 Conclusion

Lesion shape parameters can be used to differentiate between CIS converters and non-converters on a three year time scale. Our study is an example of how computational methods allow using imaging data beyond human visual analysis. In future studies, exploring the addition of other data sources, especially clinical and paraclinical features, and heading for a more differentiated prediction of the expected clinical disease course seem promising tasks.

## Declaration of interest

Haike Zhang, Esther Alberts and Viola Pongratz declare that they have no potential conflicts of interest.

Mark Mühlau: Merck, Novartis (Research Grants); Claus Zimmer: Philips, Bayer (speaker honoraria), Biogen Idec, Quintiles, MSD, Boehringer Ingelheim, Inventive Health Clinical, Advance Cor (compensation for clinical trials); Benedikt Wiestler: Bayer (speaker honoraria); Paul Eichinger: Philips Healthcare DACH (speaker honoraria), GSK foundation (travel grant).

None of the above mentioned are related to this study.
